# Editorial: Functional neuroimaging in psychiatric practice: How far have we come?

**DOI:** 10.3389/fpsyt.2023.1174588

**Published:** 2023-04-13

**Authors:** Prantik Kundu

**Affiliations:** ^1^Department of Psychiatry, Icahn School of Medicine at Mount Sinai, New York, NY, United States; ^2^Brigham and Women's Hospital, Harvard Medical School, Boston, MA, United States; ^3^Ceretype Neuromedicine Inc., Cambridge, MA, United States

**Keywords:** fMRI, connectivity, neuropsychiatric disease, artificial intelligence, diagnosis, imaging, treatment monitoring

In this Research Topic, “*Functional neuroimaging in psychiatric practice: How far have we come?*” we saw five studies applying state-of-the-art neuroimaging methodology to address diagnosis and characterization of psychiatric disease. The findings pertained diversely to ADHD, schizophrenia, bipolar disorder, treatment resistant depression, bulimia nervosa, suicidality, and the prodrome of Alzheimer's disease (AD). Functional connectivity estimated from functional MRI (fMRI) was the primary methodology in all studies. In addition, Wang et al. utilized a PET marker for Amyloid-β (Aβ) to assess the Alzheimer's endophenotype in terms of resting-state functional connectivity patterns differentiated between Aβ+ and Aβ- individuals. The study on bulimia nervosa by Li et al. used structural MRI as a correlate of functional connectivity. Demonstrating the application of functional connectivity to study the treatment effect of a novel device, Sun et al. presented resting-state fMRI evidence for Auricular Vagus Nerve Stimulation. And, representing the advanced approach of deep learning, Oliveira-Saraiva and Ferreira present cross-diagnostic predictive modeling to characterize similarities and differences between diagnostic groups.

The articles in this Research Topic recapitulate for psychiatry the precedent from neuroimaging that functional connectivity based on fMRI is essential to characterizing brain function (1). These studies demonstrate the power of this technique is in its incredible versatility for imaging diverse patient populations using the same imaging protocol. Thus, resting-state fMRI should be a component for any brain imaging strategy in neuropsychiatry. Similarly, functional MRI data generally and functional connectivity measurements specifically served as inputs to a diverse range of downstream statistical and machine learning analyses, suggesting recurring value. Notably, task-based fMRI was not represented, reflecting a trend of decreasing utility of this classical approach. This is remarkable given the importance of task-based fMRI in the first generation of fMRI study to elucidate neural correlates of function and modulation in drugs and diseases. This transition has been facilitated as an impact on psychiatry from the series of discoveries over the past decade on the correspondence of functional brain organization across task and resting states (2). With further effort, the neurobiological state of multiple domains of brain function may be clinically evaluated based on a single resting-state fMRI exam (Figure 1) to map a patient's resting-state functional networks and identify their domains of function by referencing meta-analytic atlases of functional organization based on thousands of task-fMRI studies, so that domain-associated patient networks can be evaluated with biomarkers of endogenous activity such as those described in this series to determine diagnosis or treatment response.

**Figure 1:**
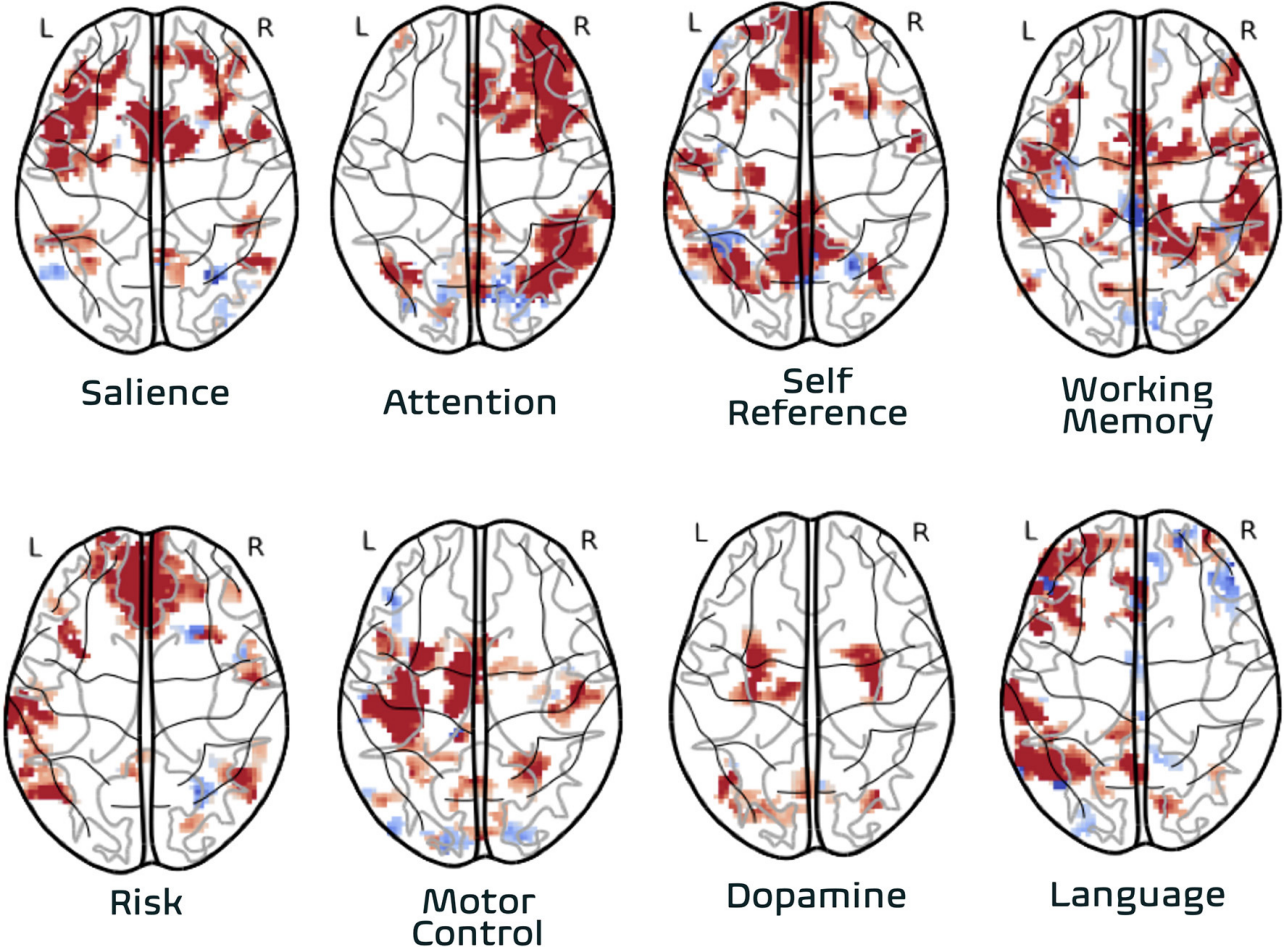
Association of individual subject functional networks to functional domains. Functional networks from one individual are derived from one resting-state fMRI dataset (8 min. acquisition) acquired with high-reproducibility fMRI strategy^∧^7,8 (multi-band multi-echo fMRI). The association to functional domains is based on automated lookup in meta-analytic database (image-based query) representing task-activation foci from over 15,000 neuroimaging studies conducted over 30 years (neurosynth.org). Association of individual subject network imaging data to functional domain made via automatically synthesized image representing functional domain using forward-inference model (frequentist) model.

In addition to estimates of inter-areal functional connectivity, resting-state fMRI data was used to characterize local functional architecture of brain tissues. The Amplitude of Low Frequency Fluctuations (ALFF) and the normalized variant fractional ALFF (fALFF) measure the amplitude of endogenous temporal signals in the frequency range most associated with resting-state fMRI. Conventionally, amplitude-based metrics of fMRI quantify the level of neural activation due to experimental task conditions; ALFF measures amplitude of neuronal activity in the absence of task (3). This measure of activity at “baseline” showed differences: in treatment resistant depression in response to auricular vagus nerve stimulation in Sun et al.; in women with past suicide attempts vs. without in Liu et al.; predictive of Aβ+ status (n.b. not cross-validated) in Wang et al.. Another use of resting-state fMRI time series data is in the estimation of local functional connectivity or regional homogeneity (ReHo) based on the computation of Kendall's [multivariate] correlation coefficient over sets of timeseries from an anatomical region (4). Wang et al. compared ALFF and ReHo in their association with Aβ+ status using a prediction framework, reporting that ReHo had moderate AUC that was statistically significant but less than AUC for ALFF.

Analytic modeling of fMRI data using learning methods (e.g., support vector machine, SVM) have been studied for several years (5). Oliveira-Saraiva and Ferreira apply a state-of-the-art artificial intelligence (AI) technique. They first use dual-regression to project a set of population-level maps of brain circuits (e.g., default mode network, motor network, etc.) onto individual subject data to produce the time series of those circuits. Then they computed the connectome among those brain circuits based on the correlations between their time series, expressed as a connectivity matrix. This produced a highly compressed representation of whole-brain connectivity for a subject, expressed in only 91 parameters. A convolutional neural network autoencoder was then used to “discover” a common set of latent rules expressed within all subject connectivity matrices. These rules are intrinsically validated by their ability to compress the data [further]. After training is complete, the rules encoded in the trained model are applied to novel datasets to compress and then decompress them. The information lost in the process reflects the accuracy of the learned model, quantified as the reconstruction error. For example, if a model were trained and/or tested with random data, reconstruction error is maximized since there are no latent patterns to train on and/or test the rules against. If effectively trained on data that share a latent pattern—even a non-linear and layered one—the reconstruction error will be lower. The power of the neural network approach is its ability to find predictive patterns more robustly than classical [regularized linear] methods such as SVM. The authors found that healthy subject data had the lowest reconstruction error, indicating a relatively high degree of consistency in brain circuitry of healthy individuals. Data of different diagnostic groups (e.g., ADHD, schizophrenia) each had significantly higher reconstruction error, suggesting paradoxically higher biological heterogeneity in patients with a common diagnosis. The convolutional neural network approach is also robust to nuisance effects by using techniques like dropout which randomly “forgets” learned parameters during training to prevent overlearning noise features (6). This was demonstrated by a diagnosis-specific model (schizophrenia) trained and tested on the dataset of one study giving similar reconstruction error when tested in separately acquired dataset. When evaluating the reconstruction error of connectivity matrices at the individual subject level, those brain circuit relationships that emerged from the average of the diagnostic group was not distinguished in individual subject connectivity, suggesting a tenuous relationship between diagnostic category and specific circuit pathophysiology.

The results presented here reflect both a capability and a need for neuroimaging to be integrated into psychiatric practice, given demonstrable capabilities in roles from diagnosis to treatment monitoring. Given the robust feasibility demonstrated here especially of resting-state fMRI, a widely accessible technique, the bottleneck of adoption of neuroimaging in psychiatry is decreasingly related to technical factors (7, 8). Thus, in the coming years, the onus will be on field of psychiatry to adopt neuroimaging techniques as part of improving care delivery toward reducing the burden of psychiatric disease on patients and healthcare systems alike.

## Author contributions

The author confirms being the sole contributor of this work and has approved it for publication.
